# BS-SCRM: a novel approach to secure wireless sensor networks via blockchain and swarm intelligence techniques

**DOI:** 10.1038/s41598-024-60338-6

**Published:** 2024-04-27

**Authors:** Jing Xiao, Chaoqun Li, Zhigang Li, Jie Zhou

**Affiliations:** College of Information Science and Technology, Shihezi University, Shihezi, 832000 China

**Keywords:** Engineering, Civil engineering, Energy infrastructure

## Abstract

In this paper, we present a novel Secure Clustering Routing Method based on Blockchain and Swarm Intelligence (BS-SCRM) for Wireless Sensor Networks (WSNs), which serves as a cornerstone in the Internet of Things (IoT) infrastructure. Recognizing the limitations of existing clustering routing methods in addressing security threats, our approach integrates blockchain technology to fortify WSNs against vulnerabilities such as man-in-the-middle attacks. The proposed BS-SCRM method is structured in two phases: (1) an enhanced cluster head (CH) election utilizing an elite strategy-enhanced Whale Optimization Algorithm (WOA) that considers node energy and proximity to the base station, and (2) a secure data on-chain phase where blockchain comes into play, encrypting and validating cluster data to safeguard integrity and prevent tampering. We further tackle the challenge of implementing blockchain in resource-constrained WSNs by assigning distinct roles to devices, i.e., ordinary nodes with data viewing permissions and accounting nodes entrusted with both data viewing and consensus algorithm execution. Extensive simulations confirm that BS-SCRM not only improves clustering quality but also provides a more secure and energy-efficient routing solution compared to contemporary methods. More specifically, simulation results in different scenarios demonstrate that BS-SCRM enhances network lifetime by 24–73% compared to other clustering methods when facing attacks.

## Introduction

WSNs serve as an essential conduit, linking the tangible physical world with the ever-growing digital sphere^1–3^. These networks are the fabric that interconnects a multitude of intelligent devices and systems into a comprehensive and complex networked ecosystem^4,5^. WSNs transcend their role as mere gatherers of data, evolving into entities that enable the intelligent exchange and consolidation of data. This facilitates the creation of a robust informational bedrock that is indispensable for intelligent analysis and the deployment of smart applications. Moreover, the synergy of WSNs with the IoT forms a dynamic intelligent infrastructure, fostering the capability for real-time data procurement^6,7^. Such immediacy in data acquisition breathes life into IoT systems, underpinning pivotal decision-making processes and automated controls across a spectrum of application areas^8–10^. For example, WSNs play a pivotal role in environmental monitoring by supplying essential data on air and water quality, as well as soil conditions. They are instrumental in smart transportation, where they contribute to traffic optimization and road safety improvements. In the realm of agriculture, WSNs enable precise monitoring of crop health and provide guidance on irrigation and fertilization, thereby enhancing agricultural productivity and produce quality.

Within the operational architecture of WSNs, clustering routing protocols are indispensable in orchestrating communication flows^1,9^. These protocols unfold through a multi-tiered process, initiating with the selection of CHs during the setup phase. The criteria for CH selection-energy levels, distance to the base station, and computational capacity-are critical for network efficiency^11^. With CHs appointed, nodes are grouped into clusters, thereby establishing a hierarchical network structure. CHs then assume the role of aggregating and relaying data from their cluster to the base station or other specified receivers^10,11^. This centralized method of data relay markedly curtails communication load and enhances energy conservation, distinguishing it from direct transmissions from nodes to the base station^12^. This layered model fosters better energy distribution and strengthens data transmission dependability, signifying a leap forward in WSN communication management.

Swarm intelligence optimization algorithms are central to the efficacy of clustering-based routing in WSNs^13–16^. Inspired by natural collective behaviors and evolutionary principles, these algorithms tackle complex issues by mimicking group cooperation and adaptive search strategies^13^. One such widely-used algorithm is WOA, which models the foraging patterns of whales^1,10,14^. Within the clustering framework of WSNs, WOA applies to pivotal tasks such as CH selection, routing path determination, and energy allocation^1,10^. The algorithm plays a significant role in boosting network performance and operational efficacy, addressing challenges like extensive data handling and energy limitations prevalent in WSNs^17^.

WSNs face numerous challenges and security risks during communication tasks^17–20^. Positioned in open wireless environments, sensor nodes are vulnerable to hazards like eavesdropping, interference, and physical tampering, which threaten the reliability and integrity of the data. Here, the decentralized nature of blockchain technology offers a robust security enhancement. It strengthens the security of clustering-based routing in WSNs by providing a safeguard against data corruption and unauthorized access^21^. Traditional clustering methods often fall short in securing data integrity and authenticity, leaving them open to various attacks. By integrating blockchain, each CH can securely log transaction data into blocks on the chain, maintaining data consistency and trustworthiness through consensus protocols. This integration marks a substantial improvement in securing the clustering-based routing operations of WSNs.

**Figure 1:**
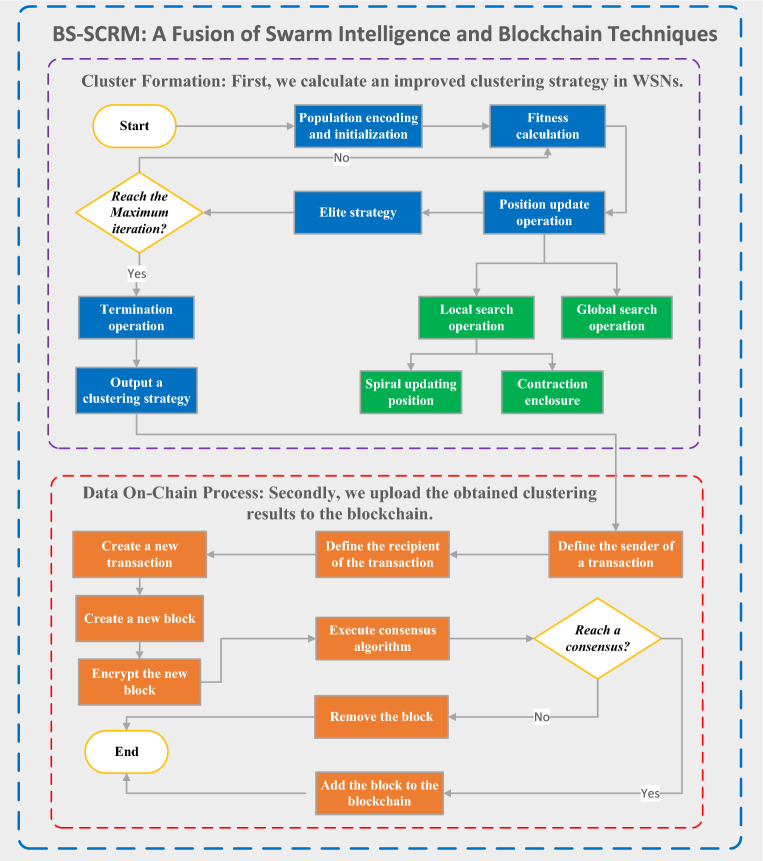
The framework of our solution.

**Motivation.** While blockchain technology presents significant promise within WSNs devices, its implementation faces several formidable challenges and constraints^22^. To begin with, the design and deployment of blockchain represent a formidable undertaking. Blockchain technology confronts intricate technical hurdles encompassing consensus mechanisms, data privacy preservation, scalability, and performance enhancement. In the context of WSNs devices, constrained by limited resources, including computational capabilities and storage capacity, the seamless integration of a comprehensive blockchain infrastructure can prove to be a daunting task. Additionally, blockchain grapples with substantial challenges regarding its performance and scalability. The fundamental architectural principles of blockchain dictate that each node must maintain complete data copies and actively participate in consensus algorithm computations, potentially resulting in diminished network performance and increased latency. To solve the aforementioned problems, we propose a novel framework, and its main structure can be illustrated in Fig. 1.

**Contributions.** This paper introduces a blockchain-based, swarm intelligence-driven Secure Clustering Routing Method (BS-SCRM) specifically tailored for WSNs. Our contributions are as follows.

- We introduced blockchain technology into the clustering routing method of WSNs, providing a more secure and energy-efficient clustering routing solution to address various security risks faced by WSNs.
- We proposed an enhanced CH election method in WSNs clustering routing, based on an elite strategy-enhanced WOA algorithm, effectively improving the quality of clustering results of BS-SCRM.
- We alleviated the challenges of applying blockchain technology to resource-constrained WSNs devices. The approach involves dividing devices in WSNs into different roles. BS-SCRM specifies that ordinary nodes have only the permission to view data recorded on the blockchain, while accounting nodes have the ability to both view data and execute consensus algorithms.
- We conducted extensive simulations to validate the effectiveness of our proposed methods and conducted comprehensive comparisons with some of the currently most representative methods of the same type.

**Roadmap.** The organizational structure of the remaining content in this paper is as follows. **Related Work** elucidates the relevant applications of WSNs clustering routing and blockchain technology. **System Model** introduces the design of a WSNs clustering routing model based on blockchain technology. Following that, **The Proposed Algorithm** delves into a secure clustering routing method named BS-SCRM proposed in this paper. **Simulation and Discussion** validates the effectiveness and security of BS-SCRM through experiments. Finally, **Conclusions** provides the conclusion and future prospects of this paper.

## Related work

### Development of WSNs Clustering Routing

Low-Energy Adaptive Clustering Hierarchy (LEACH) is one of the most influential clustering protocols in WSNs, proposed by Heinzelman et al.^23^ to save the network energy. However, because of its unplanned CH selection method, some nodes deplete their energy prematurely, thereby compromising the network’s lifetime. To address this issue, the authors introduced an improved version called LEACH-centralized (LEACH-C)^24^. This method calculates the average energy of the network through a central device (BS) and elects CH nodes. LEACH-C deals with the problem of low-energy nodes being chosen as CH, thereby extending the lifetime of the Wireless Sensor Network. Although these clsssic clustering routing methods achieved certain optimization in energy consumption, they are not suitable for modern WSNs, where higher requirements for node energy and security performance are demanded.

Traditional clustering routing methods have played a significant role in improving network energy efficiency and communication quality, but they also face many challenges^25^. To this end, new approaches and technologies have been introduced in recent years, such as swarm intelligence algorithms, to further promote the development of WSNs clustering routing^13^. These algorithms draw inspiration from collective behavior and biological evolution, simulating group collaboration and optimized search to address complex problems. For example, WOA, inspired by the hunting behavior of whales, can optimize the selection of CH nodes and route paths, thus enhancing network performance and efficiency^14^. Other swarm intelligence algorithms, like Particle Swarm Optimization (PSO)^15^ and Artificial Bee Colony (ABC)^16^, have also been widely adopted in WSNs clustering routing, providing diverse optimization solutions for the network.

### The development of blockchain technology

In recent years, blockchain technology has garnered extensive attention and research, emerging as a hot topic in the field of information technology^26^. With its features of decentralization, distribution, and immutability, blockchain is believed to hold immense potential for revolutionary impacts across various domains. As the core technology of blockchain, encryption algorithms ensure data security and privacy protection. With the continuous progress and innovation in cryptography, the application of technologies such as zero-knowledge proofs, homomorphic encryption, and multi-party computation has further enhanced the security and trustworthiness of blockchain in data transmission and storage^27^.

The distributed consensus mechanism is crucial to block-chain technology, addressing trust and collaboration among nodes in a decentralized environment^28^. In the past few years, various consensus algorithms, such as Proof of Work (PoW) and Proof of Stake (PoS), have been continuously proposed and improved, providing a solid foundation for the security and reliability of blockchain^29^. Over time, new consensus mechanisms have emerged, including variations of PoS and Byzantine Fault Tolerance, further enhancing the performance and scalability of blockchain^30^.

However, blockchain technology still faces challenges in scalability and performance. Traditional blockchain networks often encounter performance bottlenecks when processing large-scale transactions and data. To address this issue, researchers have proposed many improvement and optimization solutions, such as sharding, sidechains, and Lightning Network, to enhance the throughput and scalability of blockchain^31^. Additionally, emerging technologies like zero-knowledge proofs and privacy protection are providing new insights and solutions to tackle the scalability and privacy issues in blockchain.

### Development of blockchain technology applied to WSNs

Researchers have recognized that traditional node communication methods in WSNs often face challenges regarding security and trust, such as issues of man-in-the-middle attacks and data tampering. Therefore, introducing blockchain technology as a solution has the potential to enhance the security and trustworthiness of clustering routing in WSNs.

Currently, several studies have explored methods and mechanisms for applying blockchain technology to WSNs^32^. One common approach is to use blockchain as a secure distributed database for storing and verifying data during the communication process^33^. Nodes can record collected data in blocks on the blockchain, and the consensus mechanism ensures data consistency and immutability. Sensor nodes can access the blockchain to verify and obtain data, ensuring its credibility and integrity. Additionally, some research combines blockchain technology with smart contracts to achieve automated execution and verification of protocols and strategies during communication^34^. Smart contracts are programmable protocols executed on the blockchain, ensuring that nodes perform routing selection and data transmission while adhering to predefined rules. Introducing smart contracts enhances the security and controllability of node communication, reducing the impact of malicious nodes.

However, despite these progress, applying blockchain to clustering routing in WSNs still faces many challenges. Firstly, due to the limited resources of WSN nodes, including computational power, storage capacity, and energy supply, achieving lightweight blockchain operations on the nodes remains a difficult task. Secondly, as WSNs typically consist of a large number of nodes, efficient execution of the consensus mechanism in a distributed environment is also a challenge. Additionally, further research and improvement are required in the performance and scalability of blockchain technology to meet the efficient data transmission and processing needs in WSNs.

## System model

In this section, we will introduce the system model we have employed. Our model comprises four main sub-models, including the network model, energy consumption model, clustering model, and the application of blockchain.

### Network model

**Figure 2:**
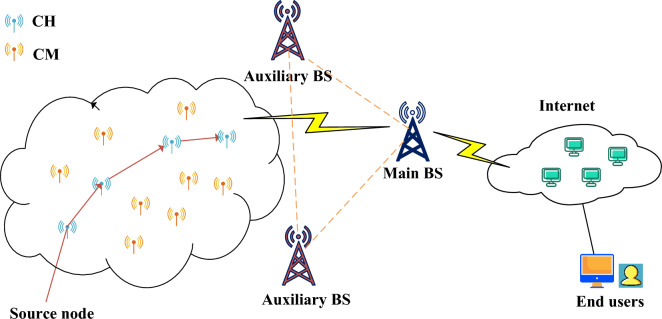
Schematic diagram of BS-SCRM.

To ensure the realism and applicability of our designed model, certain assumptions have been established: sensor nodes are uniformly and fixedly distributed within a specific area and possess identical hardware configurations. The structure of our network model, as depicted in Fig. 2, extends beyond the conventional setup of a BS and sensor nodes. It incorporates auxiliary BS nodes, vital for the integration of blockchain technology into the network. The primary BS is responsible for receiving and processing signals from various nodes, while the auxiliary BS nodes focus on verifying the data received from the main BS, thereby enhancing the network’s security and reliability.

### Energy consumption model

In WSNs, communication between nodes incurs energy consumption. Node energy consumption primarily consists of two components: one is related to radio power consumption, and the other comes from power amplifiers. The energy consumption of the former is closely related to the data transmission distance *d*. To facilitate the calculation of these energy consumptions, it is necessary to model them from a mathematical perspective. One commonly used energy consumption model for WSNs is the first-order radio model. In this model, assuming *x* bits of data are going to be sent and the data transmission distance is *d*, the transmitter’s energy consumption is given by Eq. (1).

1 $$\begin{aligned} {E_{TX}}(k,d) = \left\{ \begin{array}{l} k{E_{elec}} + k{\varepsilon _{fs}}{d^2},d < {d_0}\\ k{E_{elec}} + k{\varepsilon _{mp}}{d^4},d \ge {d_0} \end{array} \right. \end{aligned}$$

From Eq. (1), it can be observed that when *d* is bigger than ${d_0}$, the transmitter’s energy consumption dramatically increases with the communication distance. Additionally, apart from sending data, nodes receiving data also consume energy, as expressed in Eq. (2).

2 $$\begin{aligned} {E_{RX}}(x) = {E_{RX - elec}}(x) = x * {E_{elec}} \end{aligned}$$

Among them, ${E_{RX}}(x)$ denotes the energy consumption of receiving data, where *x* represents the data size, and ${E_{RX - elec}}$ is the electrical energy.

After receiving data sent by the member nodes within the cluster, the CH also needs to perform preliminary data aggregation operations to decrease the amount of data sent to the BS repeatedly. Assuming the energy consumed for data aggregation is denoted as ${E_{DA}}$, it can be calculated using Eq. (3).

3 $$\begin{aligned} {E_{DA}} = x * {\sigma _{da}} \end{aligned}$$

where ${\sigma _{da}}$ is the data aggregation factor.

### Clustering model

The process of clustering routing in WSNs typically consists of two stages: cluster formation and data stable transmission. In the former, the selection of CH nodes is of paramount importance. Nodes in this stage usually evaluate their own energy status, distance to the BS, and/or other metrics to decide whether to become the CH node. These evaluations are often based on pre-defined rules and algorithms. Nodes with lower energy consumption and closer proximity to the BS are more likely to be chosen as CH nodes. However, to ensure a balanced energy distribution throughout the network, it is common to elect nodes in a rotating way, thereby avoiding premature energy depletion in some nodes. Additionally, selecting nodes with sufficient energy and short distance to the BS as CH nodes can effectively improve data transmission efficiency as well as network performance.

In centralized clustering routing methods, the election of CH nodes is orchestrated by the BS. The BS typically decides whether a node is suitable to become a CH based on factors such as its energy status, distance, and other considerations. After selecting a set of excellent CH nodes, the remaining nodes will negotiate or compete to choose the most suitable CH node to establish a stable cluster structure. The evaluation function design for a reasonable clustering scheme in this paper is shown in Eq. (4).

4 $$\begin{aligned} Maximize \hspace{5.0pt}obj = \alpha * E + \beta * \frac{1}{D} \end{aligned}$$

where *E* represents the energy of the candidate CH node, *D* is the distance between the candidate CHs and BS, $\alpha$ is the energy factor, and $\beta$ is the distance factor.

Once a set of suitable CH election is completed, the rest of the ordinary nodes will choose the nearest CH node to join in accordance with the principle of uniform clustering. Since then, WSNs clustering has entered the stage of stable data transmission. The goal of this stage is to achieve stable and reliable data transmission through an effective data transmission strategy. Within each cluster, the CH is responsible for coordinating and managing data transmission among cluster members. Then the cluster members communicate with their corresponding CH node to transmit the collected data. CHs are responsible for aggregating, processing, and forwarding the received data, ultimately transmitting it to the BS or the target node.

To reduce energy consumption in data transmission, a commonly used strategy is Time Division Multiple Access (TDMA) scheduling. TDMA allows each cluster member to transmit data in specific time slots, thereby avoiding conflicts and interference between nodes and improving transmission efficiency.

### Application of blockchain in WSNs

To enhance the communication security of WSNs during the routing process, blockchain technology has been integrated into the system model design. Considering that IoT devices often have very limited resources, including computing, storage, and communication resources, incorporating blockchain technology into WSNs requires addressing the following issues.

***Issue 1: How to handle the resource constraints of WSNs nodes?***

**Solution:** Due to the limited computing and storage capabilities of the majority of sensor nodes in WSNs, it is challenging for them to handle the computationally intensive blockchain consensus mechanisms and maintain the complete ledger storage. One recommended approach is to divide the devices in WSNs into different roles. Therefore, the proposed model in this paper introduces two distinct role mechanisms, i.e., ordinary nodes and accounting nodes. Specifically, the sensor nodes will function as ordinary nodes within the entire network, while the BS and BS auxiliary nodes with relatively more resources will act as accounting nodes.

***Issue 2: Who is responsible for accounting in the block-chain?***

**Solution:** Based on the roles assigned to the devices in the divided WSNs, the accounting will be carried out by the BS and BS auxiliary nodes. These nodes will execute consensus algorithms that require significant computational resources and complete the process of adding blocks.

***Issue 3: How to design a blockchain consensus algorithm suitable for WSNs clustering?***

**Solution:**Unlike other classic blockchain consensus algorithms such as PoW and PoS, achieving consensus in WSNs clustering mainly depends on the election scheme for CH nodes. Specifically, the capable BS and auxiliary BS nodes will reach consensus based on the objective function defined in Eq.4.

## The proposed algorithm

This section will introduce the detailed design steps of the proposed BS-SCRM. Its main process is divided into two stages: the cluster formation stage and the data on-chain stage.

### Cluster formation method based on improved WOA

To form a well-structured cluster, a CH election scheme based on improved WOA is designed in BS-SCRM. The CH election scheme includes population encoding and initialization, fitness calculation, position update, elite operator, and termination operation.

**Population encoding and initialization:** When applying the proposed improved WOA to solve the clustering routing problem in WSNs, the first step is to determine the encoding method for individuals in the population. Since the selection of whether a node becomes a CH is a binary decision, WOA adopts a binary encoding method for individuals. Once the encoding method is established, the population in WOA needs to be initialized. The structure of the initial population is shown in Eq. (5).

5 $$\begin{aligned} Pop = \left[ {\begin{array}{*{20}{c}} {{P_{1,1}}}&{}{{P_{1,2}}}&{} \cdots &{}{{P_{1,M - 1}}}&{}{{P_{1,M}}}\\ {{P_{2,1}}}&{}{{P_{2,2}}}&{} \cdots &{}{{P_{2,M - 1}}}&{}{{P_{2,M}}}\\ \vdots &{}{}&{}{{P_{n,m}}}&{}{}&{} \vdots \\ {{P_{N - 1,1}}}&{}{{P_{N - 1,2}}}&{} \cdots &{}{{P_{N - 1,M - 1}}}&{}{{P_{N - 1,M}}}\\ {{P_{N,1}}}&{}{{P_{N,2}}}&{} \cdots &{}{{P_{N,M - 1}}}&{}{{P_{N,M}}} \end{array}} \right] \end{aligned}$$

where ${P_{n,m}} \in \{ 0,1\}$, *M* stands for the number of nodes, and *N* represents the size of population.

**Fitness Calculation:** During the optimization process of the designed WSNs clustering model, the step after population initialization is fitness value calculation. As a swarm intelligence-based algorithm, WOA requires an evaluation metric to help it select the optimal individual during the iteration process. In this case, the fitness function is the most commonly used means to assess individuals. In the proposed improved WOA, each whale individual carries a sequence of CH candidate nodes. Since the BS can obtain the remaining energy and distance of candidate CHs, the fitness of an individual can be evaluated by the fitness function Eq. (4). To illustrate, let’s provide a specific example of fitness value calculation. Assume that there are three different whale individuals ${w_1}$, ${w_2}$ and ${w_3}$ with remaining energy values of 0.7, 0.8, and 0.9 respectively, and their distances to the BS are 0.9, 0.7, and 0.5, respectively. In addition, assume that $\alpha$=0.4 and $\beta$=0.6. According to the fitness calculation formula Eq. (4), we can compute the fitness values of these whale individuals as 0.95, 1.17, and 1.56, respectively. Thus, among the three whale individuals, ${w_3}$ is relatively the optimal one in terms of fitness value.

**Position Update Operation:** To obtain an approximate optimal WSNs clustering solution, WOA updates the positions of individuals through the predation operation of the population, gradually approaching the global optimum. In the population position update of WOA, the first step is to determine the leading whale W*, which refers to the individual with the best fitness value in the current population. Once the leading whale is identified, its distance to other individuals can be calculated using Eq. (6).

6 $$\begin{aligned} d = \left| {C \cdot {W^ * }(g) - W(g)} \right| \end{aligned}$$

where *C* is the coefficient factor, *d* is the distance between two individuals, *g* represents the current number of iteration, ${{W^ * }}$ represents the position of the lead whale at generation *g*, and *W*(*g*) represents the position of the current individual at generation *g*. The position of the individual is then updated by the updated formula as shown in Eq. (7).

7 $$\begin{aligned} W(g + 1) = {W^ * }(g) - A \cdot d \end{aligned}$$

where *A* is the coefficient factor.

Coefficient factor *A* and *C* can be defined by Eq. (8) and Eq. (9) respectively.

8 $$\begin{aligned} A= & {} 2a * {r_1} - a \cdot d \end{aligned}$$

9 $$\begin{aligned} C= & {} 2 * {r_2} \end{aligned}$$

where ${r_1}$ and ${r_2}$ are random numbers between [0,1], and *a* is the control parameter and is shown by Eq. (10).

10 $$\begin{aligned} a = 2 - \frac{{2 * g}}{{{g_{max}}}}\end{aligned}$$

When $|A| < 1$, WOA performs a local search operation by imitating the bubble-net attacking behavior of whale populations. This operation can be further divided into two mechanisms: spiral updating position and contraction enclosure.

Firstly, the inspiration for the spiral updating position operation comes from the natural phenomenon of whales using a spiral motion to trap shrimp. In this behavior, whales swim in a spiral pattern towards the water surface and release bubbles to trap their prey. During this process, the distance between the leading whale ${W^ * }$ and other individuals is calculated using Eq. (11).

11 $$\begin{aligned} d' = \left| {{W^ * }(g) - W(g)} \right| \end{aligned}$$

To simultaneously simulate the spiral updating position and the whale’s contraction enclosure mechanism, the improved WOA introduces a selection probability *q*. *q* is a random number in [0,1]. If $q \ge 0.5$, the corresponding operation is given by Eq. (12).

12 $$\begin{aligned} W(g + 1) = d \cdot {e^{bl}} \cdot \cos (2\pi l) + {W^ * }(g) \end{aligned}$$

where *b* is a spiral constant, *l* is a random number in [− 1,1], *e* is the natural constant.

WOA alleviates falling into local optima through random hunting behavior. Specifically, when the coefficient *A* satisfies the condition $|A| > 1$, the WOA applies the position of the individual’s companion to achieve a position update process across the entire global scope of the problem. The formulas involved in this process are shown in Eq. (13) and Eq. (14).

13 $$\begin{aligned} d= & {} \left| {C \cdot {W_r} - W(g)} \right| \end{aligned}$$

14 $$\begin{aligned} W(g + 1)= & {} {W_r} - A \cdot d \end{aligned}$$

where ${W_r}$ is the location of a randomly selected individual whale.

**Elite Operator:** In the process of searching for a desirable WSNs clustering scheme, this paper introduces an elite strategy to accelerate the convergence speed of WOA. The original WOA is prone to losing the optimal solution generated in the current iteration, thereby affecting the convergence performance of the algorithm. To address this issue, this paper constructs an elite pool to store the fittest individuals, known as elite individuals, from each generation of the population. The implementation of the elite operator in the improved WOA involves the following four steps.

- **Step 1.** First, compute the fitness values of the current population using Eq. (4).
- **Step 2.** Find the individual with the best fitness performance in the current population and store it in the pre-constructed elite pool.
- **Step 3.** At the end of each iteration, randomly select an elite individual from the elite pool.
- **Step 4.** At the beginning of the next iteration, directly include the selected elite individual from the elite pool into the initial population to avoid losing its position during the iteration process due to the position update operation.

**Termination procedure:** If the algorithm’s iteration count reaches the specified maximum value, the algorithm terminates and provides the CH election scheme carried by the leading whale; otherwise, the algorithm returns to the step fitness calculation and continues.

After obtaining a set of CHs optimized using the improved WOA, the remaining nodes choose to join the corresponding CHs based on the principles of uniform clustering and proximity.

### Data on-chain process

We have divided the devices in WSNs into different roles for their application in the blockchain process. According to the BS-SCRM protocol, ordinary nodes are granted only the privilege to access and view the data recorded on the blockchain, while accounting nodes (i.e., BS and auxiliary BS nodes) have both the capability to access data and execute consensus algorithms, with the main BS additionally possessing the ability to validate blocks and complete data on-chain.

Based on the received energy information from sensor nodes, the accounting nodes, i.e., the main BS and auxiliary BS nodes, run the CH selection algorithm based on the improved WOA and obtain their respective sets of CH results. The information in the blocks includes the output CH sets obtained by the accounting nodes and the current energy values of all sensor nodes in the network. The blockchain consensus mechanism designed in BS-SCRM depends on the fitness values of the obtained CH sets. Specifically, it stipulates that all nodes reach consensus when confirming a set of relatively optimal fitness values for the CH set.

**Figure 3:**
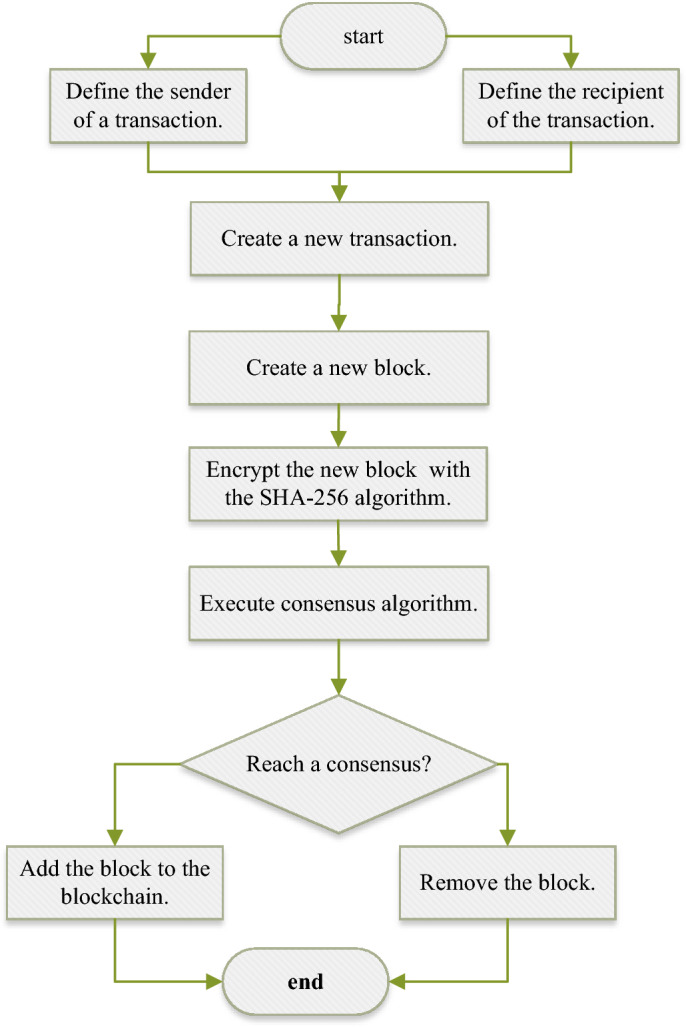
Flowchart of blockchain construction.

According to the principles of blockchain technology, each accounting node will produce a block in each round of the WSNs clustering process. However, whether this block can eventually be added to the blockchain depends on whether the recorded CH set has the optimal fitness value. In addition, in BS-SCRM, the main BS is responsible for verifying blocks generated by different accounting nodes. Also, it completes the verification of different blocks and notifies other accounting nodes of the results of the selected optimal block. These nodes need to verify whether the fitness values of the CH set sent by the main BS and the current energy values of the nodes are consistent and accurate. If they are, they respond to the main BS with confirmation messages, and then the main BS performs the data on-chain operation. Thus, a new block is added to the blockchain used in the WSNs application. Figure 3 is a flowchart for adding a blockchain to WSNs.

## Simulation and discussion

To demonstrate the effectiveness of the proposed BS-SCRM, we compared it with several similar WSNs clustering protocols, including ModifyGA^35^, EFCR^36^, and LDIWPSO^37^. Each clustering method operates in rounds, with each round consisting of a TDMA-based data collection process and a data aggregation process executed by CH nodes. The entire experimental section is divided into two subparts, namely network energy consumption testing and security performance testing. The network and algorithm parameters used throughout the experiments are presented in Table 1. It should be noted that parameters $\alpha$ and $\beta$ were determined through multiple simulations to achieve optimal performance for the proposed WOA-based CH method. Additionally, all simulations were conducted on a machine with an AMD Ryzen 9 7945HX CPU, using MATLAB R2021b software. To comprehensively evaluate all participating clustering routing algorithms, we set up four different simulation scenarios, as detailed in Table 2.

**Table 1:** Simulation parameters used in this paper.

Parameter	Value
Node initial energy	0.5 J
Packet size	4000 bits
Control packet size	200 bits
BS location	(50,150)
*P*	0.1
${g_{max}}$	40
*n*	20
*R*	4000
${\sigma _{da}}$	5 nJ/bit
${\varepsilon _{fs}}$	10 $\text{pJ}/\text{b}/{\text{m}^2}$
${\varepsilon _{mp}}$	0.0013 $\text{pJ}/\text{b}/{\text{m}^2}$
${E_{Tx}}$	50 nJ/bit
${E_{elec}}$	50 nJ/bit
$\alpha$	0.3
$\beta$	0.7

**Table 2:** Experimental scenario settings.

	Number of nodes	Node layout scene size
Scenario 1	50	100 m*100 m
Scenario 2	80	120 m*120 m
Scenario 3	100	150 m*150 m
Scenario 4	120	180 m*180 m

### Data availability

The datasets used during the current study available from the corresponding author on reasonable request.

### Network energy consumption testing

In the first testing phase, the test includes variations in the number of surviving nodes, the round at which the first node dies, and changes in the overall network’s remaining energy.

**Figure 4:**
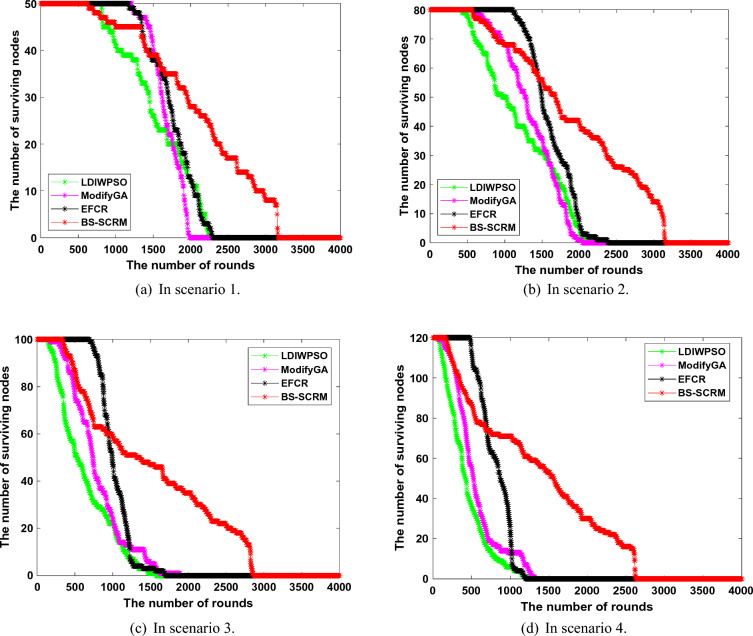
Changes in the number of living nodes.

Figure 4 shows the changes in the number of WSNs surviving nodes based on four different clustering algorithms. It can be seen that although the clustering method based on BS-SCRM has earlier rounds of dead nodes than EFCR at the initial stage of network operation, it maintains good node survival in the later network operation, which confirms that BS-SCRM is better than other algorithms in extending network lifetime. In general, from the scenario 1 to scenario 4, the maximum number of rounds of the four algorithms decreases as the number of nodes increases. Specifically, in scenario 1, although ModifyGA and EFCR appear to have dead node rounds later than BS-SCRM, their number of alive nodes is surpassed by BS-SCRM by round 1560. In the end, the three comparison algorithms basically had no surviving nodes at about 2,400 rounds, while BS-SCRM eventually had a network lifetime of more than 3,000 rounds. In the remaining three scenarios, although the comparison algorithm EFCR has a good survival node situation in the initial stage of the network, due to the limitations of the fuzzy metrics used to select CH nodes, EFCR has a rapid node death problem in the later stage of the network. In four different scenarios, the network node survival situation of BS-SCRM is much better than that of the other three comparison methods, which fully indicates that the improved WOA-based BS-SCRM obtains a desirable CH scheme.

**Figure 5:**
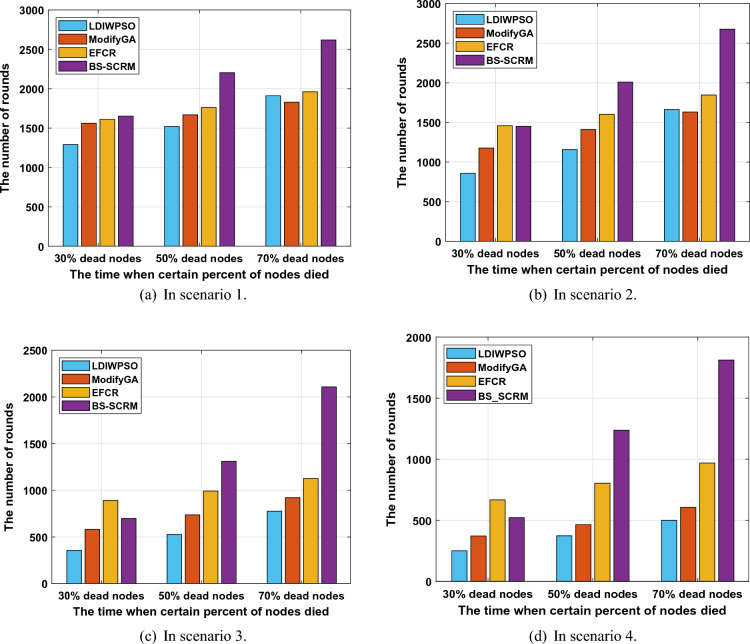
Network lifetime comparison when a certain percentage of dead nodes occur.

Figure 5 shows the comparison of the four clustering methods in different scenarios when a certain proportion of dead nodes occurs. It can be found that the network lifetime of the BS-SCRM-based clustering method is generally better than that of the other three comparison algorithms. In scenario 1, when a 50% dead node ratio occurs, BS-SCRM runs 684, 535, and 442 more rounds than LDIWPSO, ModifyGA, and EFCR, respectively. A similar situation occurred in the remaining three different scenarios. It should be noted that EFCR in the comparison algorithms is relatively optimal most of the time In 30% dead nodes, but BS-SCRM has the best performance In 50% dead nodes and 70% dead nodes. In scenarios 2, 3, and 4, BS-SCRM obtained an average of 164.9%, 130.66%, and 73.04% higher network lifetime than LDIWPSO, ModifyGA, and EFCR, respectively. This phenomenon is due to the fact that the fitness function designed by BS-SCRM comprehensively considers the factor of node energy and BS distance. Ovearll, the clustering method based on BS-SCRM has more desirable network lifetime than the other three comparison methods when there is a certain proportion of dead nodes.

**Figure 6:**
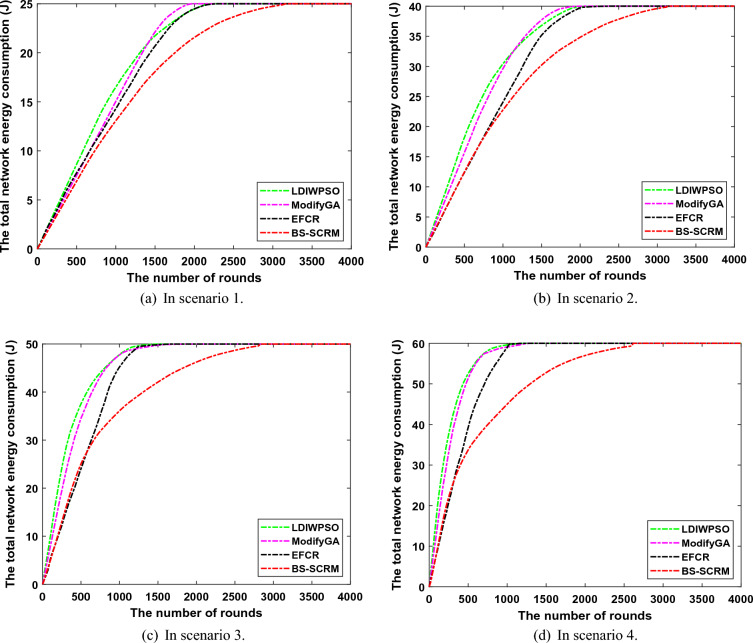
Changes in the number of living nodes.

Figure 6 compares the relationship between the total network energy consumption of WSNs and network runtime. Overall, the energy consumption based on BS-SCRM is not much different from the other three comparison algorithms in the early stage, but the energy consumption in the later stage is much lower than them. Specifically, in scenario 1, the total energy consumption of BS-SCRM when the network runs to round 500 is 6.86J, while the network energy consumption of LDIWPSO, ModifyGA, and EFCR is 8.60J, 7.45J, and 7.71J, respectively. From this, it can be seen that the energy difference in the early stage of the four different clustering methods is small. However, when the network ran to round 1500, BS-SCRM consumed 3.65J, 3.98J and 2.61J less energy than LDIWPSO, ModifyGA and EFCR, respectively. A similar trend existed in the remaining three different scenarios. Especially with the increase of the number of nodes and the increase of node layout scenarios, for example, in the 1000th round of scenario 4, the energy consumption of LDIWPSO, ModifyGA and EFCR is 14.67J, 14.15J and 13.89J higher than that of BS-SCRM, respectively. Part of the reason why BS-SCRM achieves better network power consumption control is that it uses an elite strategy to improve the traditional WOA, thus finding a better CH election scheme. This figure shows that the clustering method based on BS-SCRM has good performance in the overall energy control of WSNs.

### Security validation

In security verification of different clustering routing algorithms, the main attack is BS clustering result tampering. Specifically, this attack refers to the BS being attacked by malicious nodes and manipulated by the other side, thus changing the clustering result obtained from the normal calculation and affecting the operational stability of the network.

**Figure 7:**
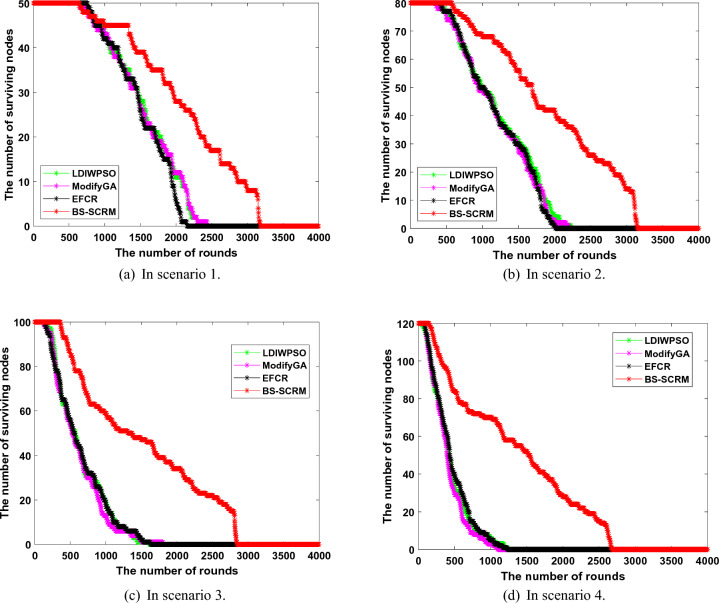
Change of the number of live nodes after BS is attacked.

Figure 7 demonstrates the change curve of network surviving nodes when the results of different clustering methods in that BS are tampered with by malicious nodes. On the whole, the node survival of BS-SCRM is not much different from that before it is attacked, while the performance of the other three comparison algorithms is significantly reduced. Specifically, before the attack, the total round of BS-SCRM nodes in four different experimental scenarios was 3166, 3154, 2860 and 2621 respectively, while after the clustering result tampering attack, the rounds of BS-SCRM was 3179, 3145, 2844 and 2679 rounds respectively. From this, it can be found that the performance of BS-SCRM is basically close to the level before the attack, thanks to its use of blockchain technology to ensure the consistency of the clustering results. In contrast, the number of surviving nodes decreased more rapidly after an attack in the other three comparison methods. For example, in scenario 3, the total node death rounds for LDIWPSO, ModifyGA, and EFCR are 8.47%, 20.43%, and 3.9% faster, respectively. In addition, the difference between these three comparison algorithms is also obvious when the number of living nodes changes. It can be seen that blockchain-based BS-SCRM has more robustness in resisting clustering result tampering attacks, while other existing clustering methods without certain security measures have the risk of premature node death.

## Conclusions

The purpose of this research is to enhance the security of WSNs routing communication and propose a secure clustering routing protocol called BS-SCRM. The protocol achieves a secure clustering routing process through the design of cluster formation and data chaining phase. Furthermore, we propose a novel CH election algorithm based on the improved WOA, which takes into consideration both node’s remaining energy and the distance to the BS in its objective function. Subsequently, we utilize blockchain technology in the data chaining phase to encrypt and verify the data of the nodes within the cluster, ensuring data integrity and tamper resistance. Experimental results demonstrate that BS-SCRM exhibits obvious advantages in terms of energy efficiency and security.

We believe that our research provides an effective solution for the security of WSNs clustering routing and offers important guidance and insights for the application and development of WSNs. Future research can further explore and optimize the BS-SCRM method and apply it in a broader range of real-world scenarios.
